# Dosimetric evaluation of four whole brain radiation therapy approaches with hippocampus and inner ear avoidance and simultaneous integrated boost for limited brain metastases

**DOI:** 10.1186/s13014-019-1255-7

**Published:** 2019-03-15

**Authors:** Aijun Jiang, Weipeng Sun, Fen Zhao, Zhenxuan Wu, Dongping Shang, Qingxi Yu, Suzhen Wang, Jian Zhu, Fengchang Yang, Shuanghu Yuan

**Affiliations:** 1 Shandong University, Jinan, 250117 Shandong China; 2 Department of Radiation Oncology, Shandong Cancer Hospital affiliated to Shandong University, 440 Jiyan Road, Jinan, 250117 Shandong China

**Keywords:** Brain metastases, Dosimetry, Hippocampus, Inner ear, Intensity-modulated radiation therapy, Volumetric-modulated arc therapy, Tomotherapy

## Abstract

**Aims:**

To perform a dosimetric evaluation of four different simultaneous integrated boost whole brain radiotherapy modalities with hippocampus and inner ear avoidance in the treatment of limited brain metastases.

**Methods:**

Computed tomography/magnetic resonance imaging data of 10 patients with limited (1–5) brain metastases were used to replan step-and-shoot intensity-modulated radiotherapy (sIMRT), dynamic intensity-modulated radiation therapy (dIMRT), volumetric-modulated arc therapy (VMAT), and helical tomotherapy (Tomo). The prescribed doses of 40–50 Gy in 10 fractions and 30 Gy in 10 fractions were simultaneously delivered to the metastatic lesions and the whole-brain volume, respectively. The hippocampal dose met the RTOG 0933 criteria for hippocampal avoidance (Dmax ≤17 Gy, D100% ≤10 Gy). The inner ear dose was restrained to Dmean ≤15 Gy. Target coverage (TC), homogeneity index (HI), conformity index (CI), maximum dose (Dmax), minimum dose (Dmin) and dose to organs at risk (OARs) were compared.

**Results:**

All plans met the indicated dose restrictions. The mean percentage of planning target volume of metastases (PTVmets) coverage ranged from 97.1 to 99.4%. For planning target volume of brain (PTVbrain), Tomo provided the lowest average D2% (37.5 ± 2.8 Gy), the highest average D98% (25.2 ± 2.0 Gy), and the best TC (92.6% ± 2.1%) and CI (0.79 ± 0.06). The two fixed gantry IMRT modalities (step and shot, dynamic) provided similar PTVbrain dose homogeneity (both 0.76). Significant differences across the four approaches were observed for the maximum and minimum doses to the hippocampus and the maximum doses to the eyes, lens and optic nerves.

**Conclusion:**

All four radiotherapy modalities produced acceptable treatment plans with good avoidance of the hippocampus and inner ear. Tomo obtained satisfactory PTVbrain coverage and the best homogeneity index.

**Trial registration:**

Clinicaltrials.gov, NCT03414944. Registered 29 January 2018

## Introduction

Brain metastases occur in 20–40% of cancer patients [1], and with improvements in local control and new effective systemic treatments, this incidence continues to increase [2]. The overall prognosis of cancer cases with brain metastases is poor. Whole brain radiotherapy (WBRT) is a fundamental radiation modality used specifically for patients with extensive brain metastases [3–7]. However, WBRT is associated with a short local control time and side effects including neurocognition dysfunction [8] and hearing deficits [9]. It is known that radiation-induced damage to the hippocampus plays an important role in the cognitive dysfunction [4, 8, 10–12], and hearing impairment can be linked to damage to the inner ear [13]. The threshold cochlear dose for hearing loss with cisplatin-based chemotherapy and radiotherapy was predicted to be 10 Gy [14]. Encouragingly, the results of the RTOG 0933 trial have shown that hippocampus avoidance can effectively reduce the cognitive impairment caused by WBRT [8].

WBRT with simultaneous integrated boost (SIB) for brain metastases has been proven to be advantageous in prolonging local control time and overall survival (OS) with a short treatment time [15, 16]. With the development of advanced radiation technologies such as step-and-shoot intensity-modulated radiotherapy (sIMRT), dynamic intensity-modulated radiation therapy (dIMRT), volumetric-modulated arc therapy (VMAT), and helical tomotherapy (Tomo), it is possible to perform WBRT with organs at risk (OARs) avoidance and SIB for brain metastases. Ferro M et al. suggested that IMRT could deliver a SIB-WBRT treatment in patients with more than 3 brain metastases [17]. Sumit Sood et al. demonstrated the feasibility of WBRT using VMAT not only to spare hippocampus, but also significantly reduce dose to OARs, including the scalp, ear canal, cochleae, and parotid glands [18]. Tomo provides a homogeneous dose distribution to the whole brain and conformally avoids the hippocampus in a single treatment plan [19]. However, it is not clear which is the most appropriate WBRT approach with hippocampus and inner ear avoidance and simultaneous integrated boost for limited brain metastases. In the present study, a dosimetric comparison between sIMRT, dIMRT, VMAT and Tomo was performed to address the technical advantages of the four treatment modalities.

## Materials and methods

### Simulation and contouring

Ten consecutive patients with 1–5 brain metastases confirmed by magnetic resonance imaging (MRI), who were treated between September 2017 and October 2017, were included. All patients had histologic evidence of non-small cell lung cancer (NSCLC). All patients were in supine position wearing a thermoplastic mask (CIVCO, Orange City, IA, USA) before computed tomography (CT) simulation. CT scans of the entire head region were acquired using a large Bore CT simulator (Philips, Cleveland, OH, USA) with a slice thickness of 1.5 mm. The 3D e-THRIVE brain MRI axial T1-weighted sequences and double gadolinium contrast-enhanced sequences were obtained with a Philips Ingenia 3 .0T MRI scanner (Philips, Cleveland, OH, USA) after CT simulation scanning. The slice thickness for MRI scans was 1.5 mm. The CT and MRI datasets were registered in the Eclipse Version 11.0 planning system (Varian Medical Systems, Palo Alto, CA, USA) for target volume delineation.

Contouring was made using 2D brush on axial images. The gross tumor volume (GTV) was defined as the contrast-enhanced lesion. The planning tumor volume for metastases (PTVmets) was generated by adding to the GTV a 3 mm margin to correct for positional inaccuracies. The whole brain volume was contoured as the clinical target volume (CTV). The hippocampus was contoured on the MRI axial T1-weighted image sequence according to the RTOG 0933 protocol [8]. The hippocampus with a 5 mm expansion was defined as the area for hippocampal avoidance. The inner ear (cochlea, vestibule, semicircular canal) was delineated on CT bone window image datasets. To construct the planning target volume for brain (PTVbrain), a 3 mm margin was added to the CTV and adjusted to not overlap the inner ear volume. Then, the area for hippocampal avoidance and the GTV volume plus 5 mm (area for dose fall-off) were subtracted from the whole brain volume. Other OARs were outlined, including optic nerves, eyes, lenses, and brainstem. All contours were delineated by the same radiation oncologist and peer-reviewed.

### Prescription and planning

The prescription dose was 45–50 Gy in 10 fractions to the PTVmets and 30 Gy in 10 fractions to the PTVbrain simultaneously. The 30 Gy in 10 fractions scheme is a common scheme used in WBRT [1, 20]. The equivalent dose in 2 Gy/fraction (EQD2) of our fractionation scheme for PTVmets was calculated to be 54.4–62.5 Gy_10_ based on the LQ model [21]. All plans aimed to encompass at least 95% of PTVmets by 100% of the prescribed dose.

We constrained the unilateral or bilateral hippocampi (subgranular zone) dose according to findings of the RTOG 0933 trial [8]. The maximum dose (Dmax) and minimum dose (D100%) limit for the hippocampal structures were 17 Gy and 10 Gy in 10 fractions, respectively. In two cases the metastasis was located close to the ipsilateral hippocampus (< 1.5 cm), we met the dosimetric constraint criteria for the contralateral hippocampus. The mean dose (Dmean) constraints for inner ear structures were 15 Gy in 10 fractions. For other OARs, the maximal doses allowed were 9 Gy in 10 fractions for lens, 37.5 Gy in 10 fractions for brainstem and optic nerves, and 40 Gy in 10 fractions for normal brain. PTVmets coverage was the first aim in the planning process. Achieving homogenous and conformal PTVbrain dose distribution without exceeding the dose constraints of OARs was the second aim. The PTVbrain coverage could be reduced to 95% or even < 90% to meet the restrictions to the OARs.

For each case, three separate treatment plans (sIMRT, dIMRT, and VMAT) were optimized with 6 MV photon beams for a linear accelerator (Trilogy, Varian Medical Systems) in the Eclipse Version 11.0 planning system. This linear accelerator is equipped with Millenium 120 multi-leaf collimator. The width of the central leaf pairs is 0.5 cm. A Analytical Anisotropic Algorithm algorithm was used for dose calculation with a spatial resolution of 2.5 mm. Beam arrangement for sIMRT and dIMRT was done using nine coplanar fields with a collimator angle of 0°. Two 360° non-coplanar full arcs were used in the VMAT plans. Tomo planning was performed using the Tomotherapy treatment planning system (Accuray® Planning Station, Hi Art® Version 5.1.3, Madison, WI, USA). Compared to 2 .5cm, 1 .0cm field width significantly improved the the whole brain homogeneity by 32% [18]. So, The field width and pitch were chosen as 1.05 cm and 0.5 cm, respectively. Each plan was calculated to achieve the optimal deliverable plan with an acceptable target coverage while not exceeding the dose constraints to OARs. All plans were designed by the same medical physicist who had 5 years of planning experience and reviewed by other experts.

### Dosimetric evaluation

Plans were finally reviewed and analyzed based on dose–volume histogram (DVH). For each patient, the target coverage (TC), conformity index (CI), and homogeneity index (HI) for PTVmets and PTVbrain were evaluated. Subsequently, the following dose–volume parameters for OARs were calculated: 1) hippocampal maximum dose, 2) hippocampal mean dose, 3) hippocampal minimum dose, 4) inner ear mean dose, 5) eyes maximum dose, 6) lens maximum dose, 7) optic nerve maximum dose, and 8) brainstem maximum dose.

According to the conformity index reported by Paddick [22], the CI was defined as:

$$ CI=\frac{{V^2}_{Tpres}}{TV\times {V}_{pres}} $$

Where *V*_*Tpres*_ represents the volume within the target receiving a dose greater than or equal to the prescription dose. *TV* represents the target volume. *V*_*pre*s_ represents the volume receiving a dose greater than or equal to the prescription dose. The value of CI can range from 0 to 1, and values closer to 1.0 are usually considered optimal.

The HI was defined as:

$$ HI=\frac{D_{2\%}-{D}_{98\%}}{D_{median}} $$

This is the most commonly used formula in the literature [23], where *D*_*2%*_ represents the maximum dose delivered to 2% of the target volume and *D*_*98%*_ represents the minimum dose delivered to 98% of the target volume. *D*_*median*_ represents the median dose to the target volume. HI is an objective tool to indicate the uniformity of dose distribution, and values close to 0 are considered optimal.

TC was defined as:

$$ TC=\frac{V_{Tpres}}{TV}\times 100\% $$

*TC* describes the fraction of the target volume receiving at least the prescription dose. For perfect TV coverage, TC equals 100%.

### Statistical analysis

Statistical comparisons among results obtained with the four treatment plan modalities were performed in SPSS, version 18.0 (SPSS Inc., Chicago, IL, USA) using one-way analysis of variance (ANOVA) with Tukey’s multiple comparison post-hoc tests. All statistical tests were two-sided, and differences were considered to be statistically significant if *p* < 0.05.

## Results

### Patient characteristics

The characteristics of the patients are summarized in Table 1. The 10 patients had a total of 26 metastases with a mean GTV of 12.8 cc (range, 1.5–79.4 cc). The lesion diameters ranged from 1.2 cm to 6.4 cm. The mean right and left hippocampus volumes were 2.97 cc (range, 2.1–4.4 cc) and 2.65 cc (range, 2.3–3.3 cc), respectively. In eight patients, lesions were located 1.5 cm away from the hippocampus. However, A metastatic lesion was in close proximity to or involved the hippocampus in two patients. The whole brain volume ranged from 1145.1 cc to 1656.0 cc. The prescription dose was 45–50 Gy in 10 fractions for PTVmets. According to the biological equivalent dose (BED) formula, the EQD2 for PTVmets prescription dose ranged from 54.4 Gy to 62.5 Gy.

**Table 1:** Characteristics of 10 NSCLC patients with brain metastases

Patient No.	Number of metastases	Total GTV volume (cc)	Bilateral hippocampus volume (cc)	Bilateral inner ear volume (cc)	Brain volume (cc)
1	2	3.7	6.3	2.9	1189.6
2	1	12.9	5.7	2.0	1339.6
3	1	6.4	4.4	2.6	1656.0
4	4	9.0	5.8	2.0	1303.4
5	3	3.2	4.7	2.6	1239.4
6	1	7.3	5.0	2.3	1478.3
7	2	79.4	2.2(R)	1.8	1335.7
8	3	1.5	6.3	2.0	1483.8
9	4	1.6	7.7	2.3	1387.2
10	5	3.3	2.7(L)	2.3	1145.1

*GTV* Gross tumor volume, *R* right, *L* left

### Dose distribution

**Fig. 1:**
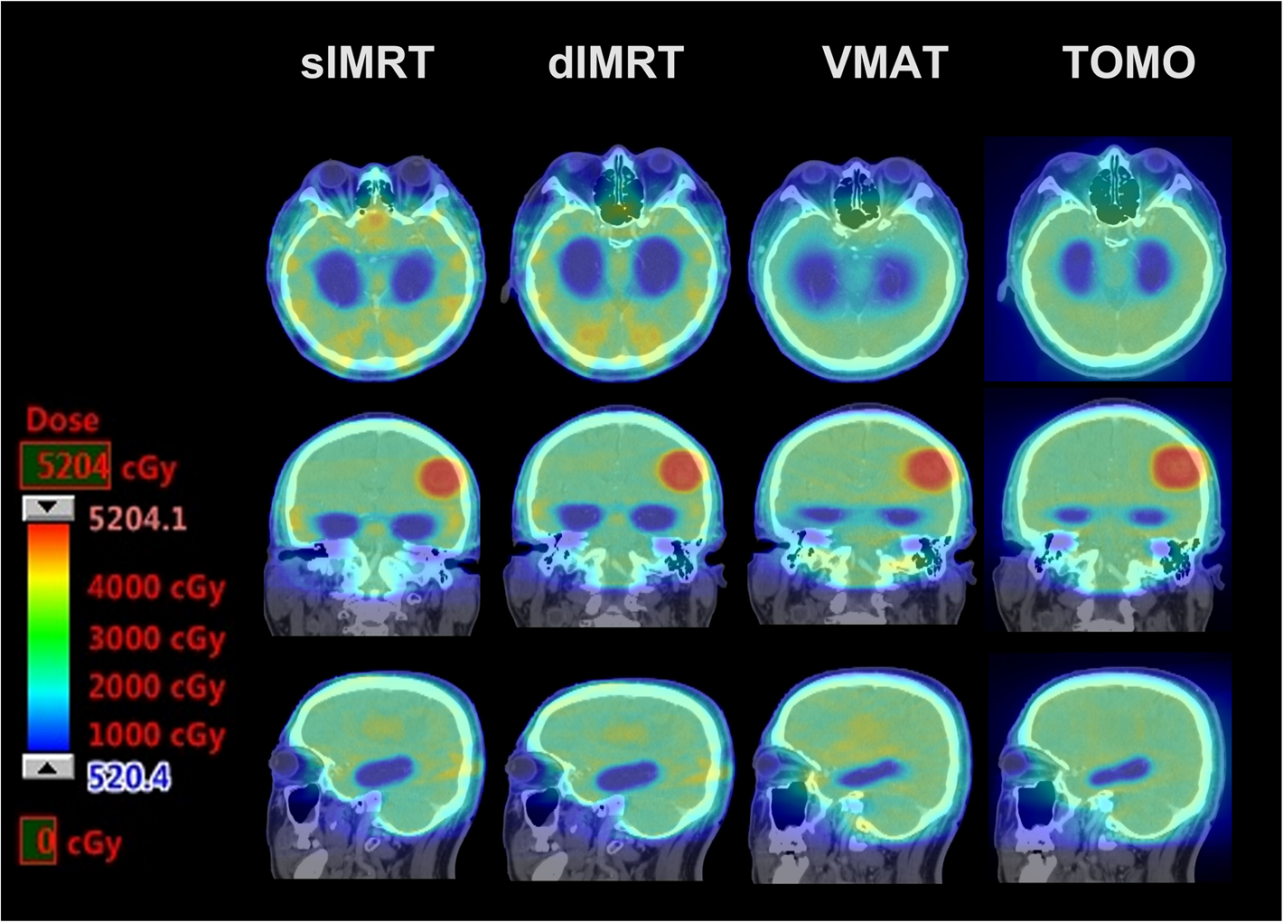
Color wash comparison of dose distributions for four modalities in a representative patient. sIMRT: step-and-shoot intensity-modulated radiotherapy, dIMRT: dynamic intensity-modulated radiation therapy, VMAT: volumetric-modulated arc therapy, Tomo: helical tomotherapy

**Fig. 2:**
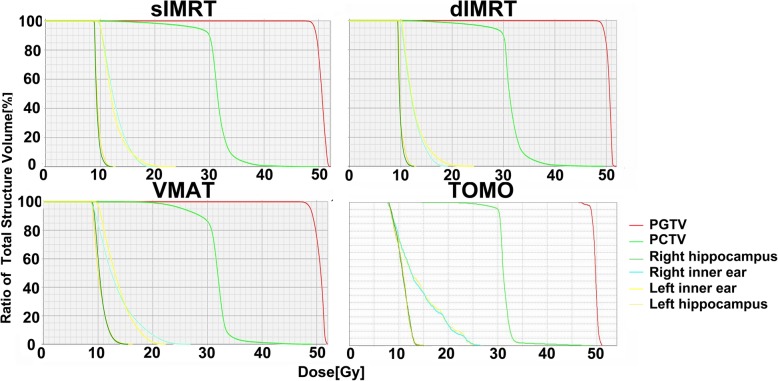
Dose–volume histograms for treatment planning with the four modalities for a representative patient. sIMRT: step-and-shoot intensity-modulated radiotherapy, dIMRT: dynamic intensity-modulated radiation therapy, VMAT: volumetric-modulated arc therapy, Tomo: helical tomotherapy

The dose parameters in the 10 patients are presented as mean values ± standard deviation (SD) in Tables 2 and 3. The prescribed dose was 45–50 Gy in 10 fractions for the PTVmets and 30 Gy in 10 fractions for PTVbrain simultaneously. Figure 1 shows a color wash representation of the dose distributions for the four treatment modalities from one representative case in our study. Figure 2 shows DVHs for the four treatment modalities from the same case.

**Table 2:** Average dose parameters for PTVmets according to the four planning modalities

	sIMRT	dIMRT	VMAT	Tomo	*p* value
D2% (Gy)	50.8 ± 2.1	50.9 ± 2.4	51.4 ± 2.7	50.2 ± 2.7	> 0.05
D98% (Gy)	47.3 ± 2.5	47.6 ± 2.4	46.8 ± 2.2	46.9 ± 2.3	> 0.05
Dmedian (Gy)	49.7 ± 2.5	49.7 ± 2.5	49.7 ± 2.5	49.3 ± 2.6	> 0.05
TC (%)	99.1 ± 1.0	99.4 ± 0.7	98.9 ± 1.6	97.1 ± 1.7	c,e,f
HI	0.071 ± 0.021	0.066 ± 0.012	0.091 ± 0.024	0.065 ± 0.023	b,d,f
CI	0.74 ± 0.10	0.73 ± 0.10	0.73 ± 0.13	0.71 ± 0.10	> 0.05

Each value was calculated from the data for all 10 patients. Values are expressed as mean ± SD. *sIMRT* step-and-shoot intensity-modulated radiotherapy, *dIMRT* dynamic intensity-modulated radiation therapy, *VMAT* volumetric-modulated arc therapy, *Tomo* helical tomotherapy. a: sIMRT vs dIMRT, b: sIMRT vs VMAT, c: sIMRT vs Tomo, d: dIMRT vs VMAT, e: dIMRT vs Tomo, f: VMAT vs Tomo. If c is in the “p value” column, it means that there is statistical significance between sIMRT and Tomo

**Table 3:** Dose parameters for the PTVbrain according to the four planning modalities

	sIMRT	dIMRT	VMAT	Tomo	p value
D2% (Gy)	39.1 ± 1.2	39.4 ± 1.1	40.6 ± 2.4	37.5 ± 2.8	e,f
D98% (Gy)	14.7 ± 3.2	14.9 ± 3.3	21.7 ± 1.1	25.2 ± 2.0	b,c,d,e,f
Dmedian (Gy)	32.1 ± 0.7	32.1 ± 0.6	33.2 ± 1.1	31.6 ± 0.4	b,d,f
TC (%)	88.2 ± 4.2	90.6 ± 1.8	88.7 ± 2.6	92.6 ± 2.1	*p* = 0.05
HI	0.76 ± 0.11	0.76 ± 0.12	0.57 ± 0.08	0.39 ± 0.11	b,c,d,e,f
CI	0.75 ± 0.04	0.77 ± 0.03	0.76 ± 0.03	0.79 ± 0.06	> 0.05

Each value was calculated from the data for all 10 patients. Values are expressed as mean ± SD. *sIMRT* step-and-shoot intensity-modulated radiotherapy, *dIMRT* dynamic intensity-modulated radiation therapy, *VMAT* volumetric-modulated arc therapy, *Tomo* helical tomotherapy. a: sIMRT vs dIMRT, b: sIMRT vs VMAT, c: sIMRT vs Tomo, d: dIMRT vs VMAT, e: dIMRT vs Tomo, f: VMAT vs Tomo. If c is in the “p value” column, it means that there is statistical significance between sIMRT and Tomo

### Dose for PTVmets

PTVmets dose parameters (mean ± SD) for the different modalities are presented along with the results of the statistical analysis in Table 2. The dose constraint was chosen as 100% of the prescription dose covering 95% of PTVmets. The D2%, D98%, Dmedian, TC, HI, and CI for PTVmets were compared. The mean percentage of PTVmets TC ranged from 97.1 to 99.4%. The HI value for VMAT was highest among those of the tested modalities (*p* < 0.05). The four planning modalities provided similar mean CIs (range, 0.71–0.74), and no significant differences in the D2%, D98%, and Dmedian were found across the modalities.

### Dose for PTVbrain

The results of the statistical dose evaluation for PTVbrain using sIMRT, dIMRT, VMAT and Tomo are shown in Table 3. Comparing PTVbrain dose distribution across modalities, Tomo provided the lowest average D2% (37.5 ± 2.8 Gy) and the highest average D98% (25.2 ± 2.0 Gy). According to the HI formula, the best target dose homogeneity would be achieved by Tomo (0.39, *p* < 0.05), followed by VMAT (0.57, p < 0.05). The two fixed gantry IMRT modalities (step and shot, dynamic) provided similar dose homogeneity (both 0.76). Although special care was taken to ensure that there was no dose larger than 120% of the prescription dose within the PTVbrain, some hotspots (Dmax > 36 Gy) were still found with the four planning modalities. These were considered acceptable though due to their small volume which limits their clinical significance (not D2%). No statistically significant differences in TC and CI were found across modalities.

### Hippocampus and inner ear sparing

The dose parameters of hippocampus and inner ear are compared in Table 4. All plans met the RTOG 0933 protocol dose compliance criteria for hippocampal sparing. Significant differences in Dmax and Dmin were found across the four planning modalities (p < 0.05). There are no statistical significance for mean doses between sIMRT, dIMRT, VMAT and Tomo. Compared to VMAT and Tomo, sIMRT and dIMRT yeilded lower average maximum doses to the hippocampus. The lowest average minimum doses (D100%) to the hippocampus was achieved by Tomo plan.

**Table 4:** Doses to OARs

	sIMRT	dIMRT	VMAT	Tomo	p value
Hippocampus Dmax (Gy)	14.3 ± 1.1	14.5 ± 1.4	15.8 ± 0.7	15.5 ± 0.9	b,c,d,e
Hippocampus Dmean (Gy)	9.7 ± 0.2	10.1 ± 0.3	10.0 ± 0.6	10.0 ± 0.7	> 0.05
Hippocampus Dmin (Gy)	8.9 ± 0.3	9.3 ± 0.2	8.3 ± 0.4	7.6 ± 0.7	a,b,c,d,e,f
Left inner ear Dmean (Gy)	13.7 ± 0.7	13.8 ± 0.8	13.4 ± 1.0	13.1 ± 1.0	> 0.05
Right inner ear Dmean (Gy)	13.6 ± 0.8	13.8 ± 0.7	13.7 ± 1.6	13.3 ± 1.1	> 0.05
Eyes Dmax (Gy)	32.5 ± 4.9	32.9 ± 4.8	26.7 ± 6.1	30.8 ± 4.7	b,d,f
Lens Dmax (Gy)	5.7 ± 0.6	5.8 ± 0.6	6.8 ± 1.3	5.5 ± 0.5	b,d,f
Optic nerves Dmax (Gy)	33.6 ± 1.7	33.8 ± 1.7	32.0 ± 2.0	31.3 ± 0.9	c,e
Brainstem Dmax (Gy)	35.4 ± 1.1	35.7 ± 0.9	35.9 ± 0.6	34.0 ± 0.2	c,e,f

Each value was calculated from the data of all 10 patients. Values are expressed as mean ± SD. *sIMRT* step-and-shoot intenseity-modulated radiotherapy, *dIMRT* dynamic-intensity modulated radiation therapy, *VMAT* volumetric-modulated arc therapy, *Tomo* helical tomotherapy. a: sIMRT vs dIMRT, b: sIMRT vs VMAT, c: sIMRT vs Tomo, d: dIMRT vs VMAT, e: dIMRT vs Tomo, f: VMAT vs Tomo. If c is in the “p value” column, it means that there is statistical significance between sIMRT and Tomo

The entire auditory-vestibular system is vulnerable to RT injury. Therefore, we constrained the inner ear dose to Dmean ≤15 Gy. Table 4 also summarizes the mean inner ear doses for the four plan modalities. There were no significant differences across modalities.

### Doses to other OARs

For eyes, lenses, optic nerves and brainstem, structures not specifically mentioned in the RTOG 0933 protocol dosimetric compliance criteria, all four techniques met the dose constraints we set. The average maximum doses are compared in Table 4. Tomo yielded the lowest average maximum doses to lenses, optic nerves and brainstem. The highest average maximum dose in lenses was achieved by the VMAT plan, and the difference was significant. These differences are attributable to the plans with two full non-coplanar arcs.

## Discussion

WBRT is associated with short local control and side effects, including neurocognition dysfunctions [8] and inner ear deficits [9]. The potential radiation-induced damage to OARs have been underestimated. With the development of sIMRT, dIMRT, VMAT and Tomo, it is feasible to reduce neurocognitive toxicity and inner ear deficits by using advanced techniques. It is possible that SIB-WBRT with hippocampus and inner ear avoidance can achieve the best balance between intracranial tumor control and cognitive function protection. In the present study, we evaluated the dosimetric advantages of four SIB-WBRT radiotherapy modalities (sIMRT, dIMRT, VMAT and Tomo) with hippocampus and inner ear avoidance for the treatment of limited brain metastases. To summarize the results, while all four modalities achieved PTVmets target coverage and met the basic dosimetric compliance criteria, we found that TomoTherapy provided the most homogeneous PTVbrain target dose (mean HI = 0.39).

Recent reports on SIB-WBRT showed promising outcomes with a shorter treatment period [15, 16]. Both median intracranial progression-free survival and median survival were increased up to 10 months [24]. The SIB technique was also found to achieve good hippocampus sparing [25]. In the present study, SIB-WBRT with hippocampus and inner ear avoidance was replanned and compared. All four planning modalities provided good mean PTVmets target prescription coverage (range, 97.1–99.1%) and similar D2%, D98%, Dmedian, and CI values. The HI value for VMAT was higher than those with the other three modalities (0.091 vs 0.065–0.071, *p* < 0.05), but this difference is small and has little clinical significance considering that brain metastases are commonly surrounded by healthy brain.

For the whole brain volume, good target coverage, CI and dose homogeneity are very important. In our study, the best target dose homogeneity was achieved by Tomo (0.39, p < 0.05), followed by VMAT (0.57, p < 0.05). The two fixed gantry IMRT modalities (step and shot, dynamic) provided similar dose homogeneity (0.76, respectively). Tomo plans could achieve better dose distribution in the PTVbrain over the other techniques, as already confirmed by Cozzi et al. [26] No statistically significant differences in TC and CI were found across modalities. Thus, the four modalities yielded similar results in target volume coverage and conformity.

A phase II multi-institutional trial (RTOG 0933) showed that hippocampus avoidance during WBRT is associated with preservation of memory and quality of life in patients with brain metastases [8]. The development of advanced techniques such as VMAT and Tomo is increasing the feasibility of this treatment paradigm [27–29]. In the present study, we demonstrated that all four planning modalities (sIMRT, dIMRT, VMAT and Tomo) met the RTOG 0933 protocol dose compliance criteria for hippocampal sparing. Similar mean doses to the hippocampi were achieved with the four planning modalities with an acceptable mean PTVbrain coverage (88.2%–92.6). Compared to healthy brain, the dose to the hippocampi was lower by approximately two-thirds. The entire inner ear system is also vulnerable to RT injury, and dose reduction to this region must be considered even at the relatively low dose of WBRT, as many patients receive some form of cytotoxic therapy. We also demonstrated that the mean inner ear dose was significantly reduced to Dmean ≤15 Gy. None of the four modalities produced any notable dose difference in the dose to the brainstem, since the brainstem contour overlapped the PTVbrain volume in all cases. With the use of two full non-coplanar arcs, the average maximum dose to the lenses was highest with VMAT planning.

Although our results show that the four SIB-WBRT modalities can produce acceptable treatment plans with good avoidance of the hippocampus and inner ear, it is important to note that some limitations of the work exsist. For instance, the four investigated techniques are not only delivery techniques but also associated to treatment planning as well, in particular to plan optimization. In addition, treatments using the CyberKnife or GammaKnife are also very common for brain metastases [30, 31], which are not mentioned in the study. Moreover, only one physicist was involved in the treatment planning. This procedure could bring some bias to our result. The application of automatic treatment planning approaches promises to reduce this bias.

## Conclusions

Our data has demonstrated that all four SIB-WBRT modalities (sIMRT, dIMRT, VMAT and Tomo) can produce acceptable treatment plans with good avoidance of the hippocampus and inner ear. Tomo can provide satisfactory PTVbrain coverage with the highest HI but seems the least efficient, whereas VMAT can provide a sufficient dose distribution with remarkably reduced MUs.

## Abbreviations

BED: biological equivalent dose
CI: conformity index
CT: computed tomography
CTV: clinical target volume
dIMRT: dynamic intensity-modulated radiation therapy
Dmax: maximum dose
Dmax: maximum dose
Dmin: minimum dose
DVH: dose–volume histogram
GTV: gross tumor volume
HI: homogeneity index
MRI: magnetic resonance imaging
MUs: monitor units
NSCLC: non-small cell lung cancer
OARs: dose to organs at risk
OS: overall survival
PTVbrain: planning clinical target volume
PTVmets: planning gross tumor volume
SIB: simultaneous integrated boost
sIMRT: step-and-shoot intensity-modulated radiotherapy
TC: target coverage
Tomo: helical tomotherapy
VMAT: volumetric-modulated arc therapy
WBRT: Whole brain radiotherapy
